# Originality in online dating profile texts: How does perceived originality affect impression formation and what makes a text original?

**DOI:** 10.1371/journal.pone.0274860

**Published:** 2022-10-19

**Authors:** Tess van der Zanden, Alexander P. Schouten, Maria B. J. Mos, Emiel J. Krahmer

**Affiliations:** Department of Communication and Cognition, Tilburg School of Humanities and Digital Sciences, Tilburg University, Tilburg, The Netherlands; Universita degli Studi di Perugia, ITALY

## Abstract

This paper investigates origins and consequences of perceived profile text originality. The first goal was to examine whether the perceived originality of authentic online dating profile texts affects online daters’ perceptions of attractiveness, and whether perceptions of (less) desired partner personality traits mediate this effect. Results showed the positive impact of perceived profile text originality on impression formation: text originality positively affects perceptions of intelligence and sense of humor, which improve impressions of attractiveness and boost dating intention. The second goal was to explore what profile text features increase perceptions of profile text originality. Results revealed profile texts which were stylistically original (e.g., include metaphors) and contained more and concrete self-disclosure statements were considered more original, explaining almost half of the variance in originality scores. Taken together, our results suggest that perceived originality in profile texts is manifested in both meaning and form, and is a balancing act between novelty and appropriateness.

## Introduction

In music, movies, and books, originality may be seen as one of the best ways to someone’s heart. Not only is original work considered more attractive, as opposed to less original work, but this also generalizes to the producer of the work [1]. It was through the texts they wrote that Lord Byron, Honoré de Balzac, and Victor Hugo achieved good regard and hence conquered (many) hearts [2]. Other research on professional and non-professional writers also found that the success of writers, as for example measured by number of publications or text evaluations, can indicate writers’ success in attracting potential partners [3,4].

According to Sternberg [5], “few psychological constructs have proved more elusive to define” (p. 126) than creativity and originality, which may also explain the variation in definitions used for the constructs (e.g., [6,7]). This study uses the term (text) originality to refer to texts that are not common and differ from most others in the specific communicative setting for which they are written, with original texts being a potential result of the creative process that has taken place.

A range of methods has been used to measure originality in texts, with for example studies on prose, poetry, and song lyrics using objective measurements such as the number of unique words (e.g., [8–10]). While most of these studies found that originality can predict a product’s popularity and success, less is known about what texts people deem original, and how perceptions about a text’s originality affect impressions that are formed about writers of these texts. What specific features constitute originality in texts that are perceived as being original is also understudied. These issues are taken up in the current study, in the context of online dating profile texts, where an original text may be particularly relevant to attract potential romantic partners.

In online dating, the free-text component of dating profiles offers many opportunities to be original. An original dating profile text can be effective to attract attention, which may be particularly appealing now that the greater use of online dating increases the number of users and profiles of these people [11]. Moreover, if originality in writing is indeed evaluated positively, profiles that are perceived as original may also be seen as attractive, and owners of these profiles might then have more success in attracting potential romantic partners. Yet, there is no research that investigates how perceived originality in online dating profiles affects impression formation and how it is constituted in profile texts. To investigate this, the present study contains a perception study and a content analysis.

The goal of the perception study is to investigate the relation between perceived profile text originality, personality impressions, and attractiveness impressions. More specifically, it is examined whether the relationship between perceived profile text originality and impressions of attractiveness and dating intention is mediated by perceptions of personality traits considered more and less attractive in romantic partners, namely intelligence, sense of humor, and oddness. The participants in this study were users of web-based dating sites, with a large majority registered on a website that primarily caters to users over 50 years and relies on dating profiles on which the textual component plays a central part. They were presented with a maximum of five of a total of 308 different authentic dating profile texts and were asked to evaluate the profile (owner).

In the content analysis, we then try to identify the characteristics that are predictive of profile texts that are perceived as original. Research suggests that original texts should be different from what others write (novel) but should also be socially meaningful (appropriate; [12]), and that texts can be original in *what* is written (meaning) and/or *how* the text is written (form; e.g., [6]). For that reason, both meaning and form characteristics were included in the analyses. All profile texts of the perception study were both manually and automatically coded on a number of features that could be indicative of perceived text originality. Online daters’ originality scores given to the texts were then used to examine what characteristics resulted in increased perceptions of profile text originality.

Taken together, this study adds to the current literature on online dating, but the implications of this study may also more broadly contribute to theory on (perceived) originality in texts. Results of the perception study provide insights on whether people generally agree on what texts are original, and to what extent the perceived originality of texts affects perceptions of writers. Results of the exploratory content analysis reveal whether it is possible to identify characteristics that make a text appear original, and whether perceived text originality is a multifaceted construct that is manifested by characteristics of meaning and form.

## Perceived originality in online dating profile texts

For profile owners, dating profiles seem to have two related purposes: to display an attractive self and to catch the attention of potential partners (e.g., [11,13]). The free-text component of dating profiles, in the form of profile texts, can be used to serve either of the goals. On web-based dating sites, the profile text is often created in a section called “This is me” or “Who am I?”, in which profile owners are asked to describe themselves in their own words [14], for example by writing about their occupation, personality, favorite interests and activities, and desired relationship partner and relationship goals.

An original text on a profile is one way to attract attention and to positively affect impressions. But from both the perspective of profile owners and profile observers, originality can potentially be a concern as well [15–17]. Profile owners have indicated they struggle with balancing a desire to stand out with the need to blend in [15]. Dating profile observers, on the other side, mentioned in interview studies that the lack of originality and creativity and the (over)abundance of clichés may lead to negative attitudes towards profiles and their owners [16,17]. Various dating sites address this concern, and advise their users to write unique profile texts. For example, on their website, dating platform eHarmony [18] states “users whose profiles are heavy on the clichés tend to get fewer messages and responses than those whose profiles show thought, originality and a genuine sense of humor.”

The vast majority of profile texts still appear cliché-ridden and generic [16,17,19] and show a high level of predictability [20]. Most profile owners present similarly (selective) information in which common attributes, such as self-descriptions of being spontaneous and kind, and common interests and activities are emphasized (e.g., love to laugh and travel, like to sip wine by fireplaces, go for romantic strolls on the beach; [16,17]). To be more original in their profile text, therefore, profile owners could write texts that are novel and differ (in some way) from what is generally seen, both regarding the (personal) information that is provided and in the phrasing, word and stylistic choices that are made.

Such highly generic profiles full of clichés often lack novelty, but are simultaneously highly appropriate (i.e., they follow all conventions regarding these texts). Appropriateness is the other important dimension that influences perceptions of originality [12]. What is appropriate highly depends on shared expectations, conventions, and norms that have emerged over time in the specific context of online dating profiles, which simultaneously generates assumptions about what (linguistic) behavior is unexpected [21,22]. Following the expectation violations theory of Burgoon and Jones [23], profile texts that do not conform to existing conventions, such as those that do not contain any personal information, negatively violate (social) expectancies and norms [24]. Such unexpected behavior can, in turn, negatively affect impression formation, for instance with regard to general favorability [25]. It thus seems that the originality of a profile text may positively affect impressions that others form about the profile owner’s attractiveness, but only so long as it happens within the boundaries of appropriateness.

The positive correlation between creativity and the personality traits intelligence and sense of humor may be one of the mechanisms behind this positive effect of originality (e.g., [26,27]). It has been argued that to be creative at least a moderate level of (verbal) intelligence is necessary [28]. Research even suggests there is a substantial overlap between cognitive intelligence and creativity [26,29], with intelligent people being better at self-expression and language play [30]. Gao and colleagues [28] examined this in a dating context and found that women were more willing to date men who used metaphorical language to compliment their appearance than those using literal language (e.g., “Your eyes are shining stars” vs. “You have beautiful eyes”). Men producing metaphorical compliments did not only score higher on a verbal intelligence test, but they were also perceived as more intelligent [28].

Besides increased positive perceptions of intelligence, the perceived originality of a profile text may also enhance positive perceptions about a profile owner’s sense of humor. Online daters use humor in their profile texts as a strategy to appear unique and more creative [17]. Positive correlations have been found between perceptions of creativity and sense of humor (e.g., [26,27,31,32]). It has been suggested that since humor and creativity share many features, such as playfulness and risk taking, humor can even be seen as a subset of creativity [33]. Therefore, some level of (verbal) creativity is required to generate humor (e.g., [27,33]).

Both intelligence and humor are, in turn, important determinants when assessing the attractiveness of a potential partner (e.g., [34,35]). Also in an online dating context, it has been shown that owners of profiles that appear to be more intelligent (e.g., [36]) and humorous (e.g., [37,38]) are deemed more desirable relationship partners. In all, we pose the following two hypotheses:

**H1.** Perceived profile text originality increases perceptions of profile owners’ intelligence which, in turn, positively affect perceptions of profile owners’ attractiveness and dating intention.

**H2.** Perceived profile text originality increases perceptions of profile owners’ sense of humor which, in turn, positively affect perceptions of profile owners’ attractiveness and dating intention.

However, in the context of online dating, where presenting the (attractive) self to potential romantic partners is the foremost purpose of profiles, the appeal of coming across as original may be constrained by the need to stick to conventions: daters’ expectations about the kind of profile cues that are appropriate and meaningful have to be taken into account as well [21,22]. As such, it may be expected that writers of profile texts that do not satisfy the appropriateness criteria, for example when regular conventions are exceeded, may come across as odd. Oddness here refers to owners of profiles that score high on perceived strangeness, eccentricity, and peculiarity (e.g., [39–42] by “acting and thinking in creative and unusual ways which sets them apart from their more conventional peers” [43; p. 205).

Once a profile owner does not conform to social expectations and norms in a particular situation or context, this can guide impression formation, such as about the person’s social skills. In the online dating context, profile owners who deviate too much from others in how they textually present themselves may be evaluated as being peculiar in their way of thinking. More specifically, if a profile text deviates to such an extent that it is no longer appropriate in the dating context, this may suggest that this person also behaves distinctively in other situations, such as in face-to-face encounters or in later relationship stages. This can negatively affect the predictability of the anticipated behavior of these profile owners, which is considered uncomfortable and undesirable in dating contexts (e.g., [44,45]). Indeed, in the context of flirting behaviors in public settings (e.g., in a club), White and colleagues [46] found that the percentage of participants favoring unexpected behavior of potential romantic partners (8.11% of the participants; e.g., a person reciting Shakespeare to the participant) was lower compared to those who preferred highly expected behavior (57.12% of the participants, e.g., a person adding the participant on Instagram). This leads to the following hypothesis:

**H3.** Perceived profile text originality increases perceptions of profile owners’ oddness which, in turn, negatively affect perceptions of profile owners’ attractiveness and dating intention.

In the second part of our study, we investigate the specific characteristics that determine perceived originality in texts. Research is scarce on what exact characteristics increase perceptions of originality in dating profile texts, but previous research has highlighted that both meaning (or content) and form (or style) can determine text originality (e.g., [8,47]). De Saussure [48] was one of the first linguists who posed that in language, meaning (the “signified”) and form (the “signifier”) together convey a communicative message (the “sign”). It is expected that perceived originality in dating profile texts is also manifested through both characteristics of meaning and form.

Meaning involves the content that is provided or the concept that is represented, which also includes the (type of) topics that are discussed in a text. For example, previous research has shown that in (popular) sonnets and song lyrics characteristics such as a wide range of different topics and including highly specific and rare topics–in relation to other texts in the genre–can be indicative of originality [8,47–50], and we conjecture that comparable meaning features increase perceptions of originality in dating profile texts.

Form refers to the language style that is used to make meaning. Various forms can be used to describe specific content or a concept: “I’m looking for my other half” and “I hope to find someone to fall head over heels for” are two other forms to express “Looking for a (long-term) relationship partner”. Writers’ stylistic choices may also enhance a text’s imagery and vividness, which is another important attribute that has been associated with originality (e.g., [51,52]). Earlier research found that the popularity of original work is negatively associated with imagery of abstract ideas and concepts and positively associated with imagery of concrete experiences, sensations, and desires [47,50]. Examples of form characteristics that could evoke readers’ affect, images, and other sensory inputs are the use of more unique and concrete words, and more metaphors and other figures of speech [6,28,50]. Similar form characteristics may be observed in dating profile texts that are perceived as original.

In addition to the hypothesis-testing perception study, we also explore what specific text characteristics increase perceptions of profile text originality. To do so, we use the originality ratings given to all 308 texts in the perception study to construct a codebook with a number of features that may be indicative of perceived profile text originality. These 308 profile texts are then coded on those features. By doing so, we aim to answer the research question for the content analysis part of this study, which is: **What characteristics in online dating profile texts increase perceptions of profile text originality?**

Both the perception study and content analysis study are preregistered on the Open Science Framework (OSF; see https://osf.io/yns83/). In Spring 2020, the Research Ethics and Data Management Committee (REDC) of the school of our university provided ethical clearance to conduct both studies.

## Perception study: Effects of perceived profile text originality

### Method

#### Participants

1234 participants took part in this study, all with an account on one of the two web-based dating sites with whom we collaborated for this study. The collaborating dating sites were: 50PlusMatch, which presents itself as a dating site for active people of 50 years or older, and Pepper, a general-purpose dating site. From all participants, 1153 were members of 50PlusMatch (93.4%) and 81 of Pepper (6.6%). The participants’ mean age was 63.5 years (*SD* = 12.1) and Dutch was the native language of 96.9% of the participants. Almost half of them indicated to be female (47.2%), and 96.4% indicated to feel mostly attracted to the opposite sex. More than half of the participants had a vocational or high school level degree (54.7%), and 44.7% had obtained a college degree.

Both sites assisted with participant recruitment but were not involved in any further aspects of the study, such as the experimental setup or the study outcomes. Participation was on a voluntary basis. The participants could participate in a raffle for ten vouchers of a three-months free membership of the dating site.

#### Materials

To construct the materials for this study, 308 profile texts were selected from a sample of 31,163 dating profiles from two existing Dutch dating sites (other sites than the participants’ sites). These profiles were written by people with different ages and education levels. A large subset of the sample were profiles from a general dating site, the remainder were profiles from a site with only higher educated members (3.25%). The collection of this corpus was part of an earlier research project [53] for which we scraped in March 2017 profiles with the online tool Web Scraper and for which we obtained separate approval by the REDC of the school of our university. Only parts of profiles (i.e., the first 500 characters) were extracted, and if the text ended in an incomplete sentence because the upper limit of 500 characters had been retrieved, this sentence fragment was removed. This maximum of 500 characters also allowed use to create a sample in which text length variation was limited. For the current paper, we relied on this corpus for the selection of the 308 profile texts which served as starting point for the perception study. Texts that contained fewer than ten words, were written fully in another language than Dutch, included only the general introduction generated by the dating site, or included references to pictures were not selected for this study.

A central question in our study was what constitutes originality in dating profile texts. Because we did not know this prior to the study, we used authentic dating profile texts to construct the materials for the study instead of fictitious profile texts that we created ourselves. To guarantee the privacy of the original profile text writers, all texts used in the study were pseudonymized, which means that identifiable information was swapped with information from other profile texts or replaced by comparable information (e.g., “My name is John” became “My name is Ben”, and “bear55” became “teddy56”). Texts that could not be pseudonymized were not used. None of the 308 profile texts used for this study can thus be traced back to the original writer.

**Fig 1:**
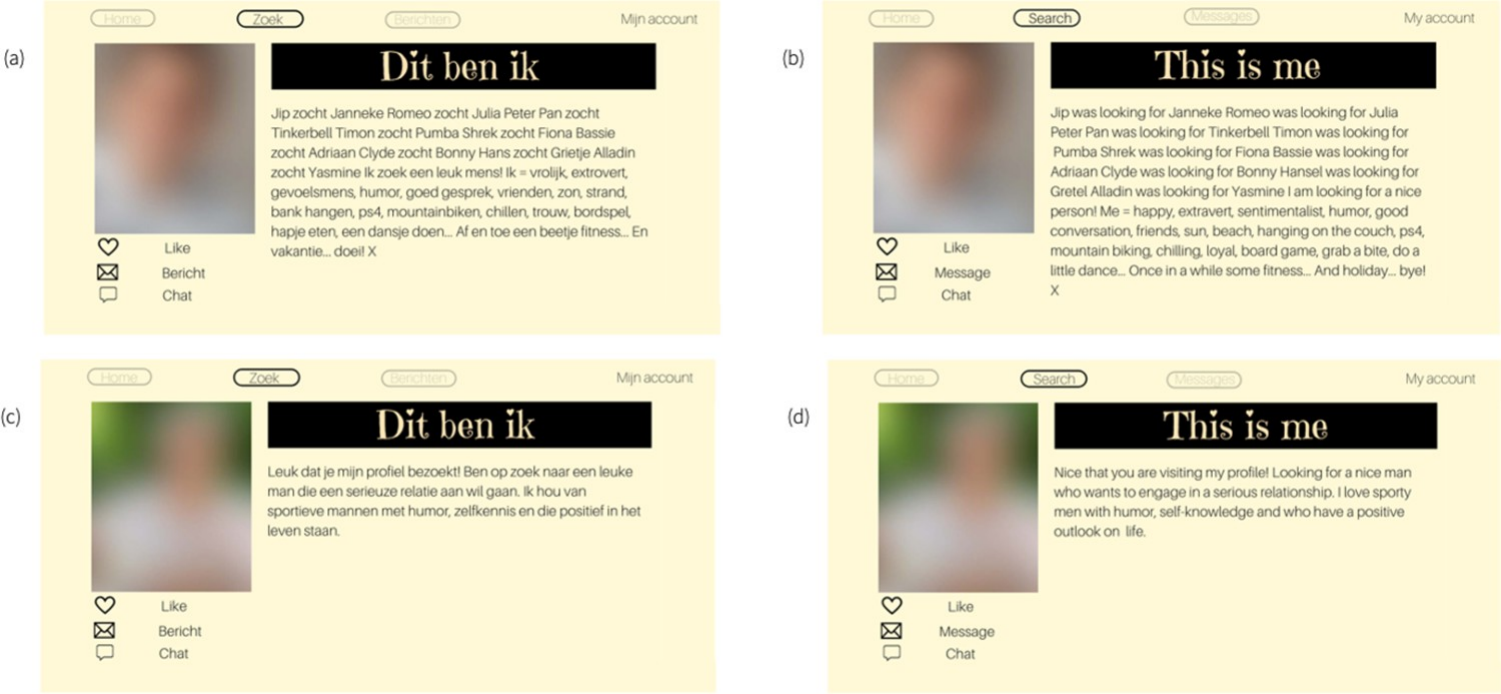
Examples of the original Dutch dating profiles used for the experiment (a, c) and their translated English versions (b, d). Profiles (a) and (b) are male profiles with a high TF-IDF score (bin seven), and (c) and (d) are female profiles with a low TF-IDF score (bin one).

A preliminary scan by the authors showed little variation in originality among the vast majority of texts from the corpus, with most texts containing fairly generic self-descriptions of the profile owner. Therefore, a random sample from the entire corpus would result in little variation in perceived text originality scores, making it difficult to examine how variation in originality scores affects impressions. As we aimed for a sample of texts that was expected to vary on (perceived) originality, the texts’ TF-IDF scores were used as an initial proxy of originality. TF-IDF, short for Term Frequency-Inverse Document Frequency, is a measure often used in information retrieval and text mining (e.g., [54]), which calculates how often each word in a text appears compared to the frequency of this word in other texts in the sample. For each word in a profile text, a TF-IDF score was calculated, and the average of all the word scores of a text was that text’s TF-IDF score. Texts with high average TF-IDF scores thus included relatively many words not found in other texts, and were expected to score higher on perceived profile text originality, whereas the opposite was expected for texts with a lower average TF-IDF score. Looking at the (un)usualness of word use is a commonly used approach to indicate a text’s originality (e.g., [9,47]), and TF-IDF seemed a suitable initial proxy of text originality. The profiles in Fig 1 illustrate the difference between texts with a high TF-IDF score (original Dutch version that was part of the experimental material in (a), and the version translated in English in (b)) and those with a lower TF-IDF score (c, translated in d).

**Fig 2:**
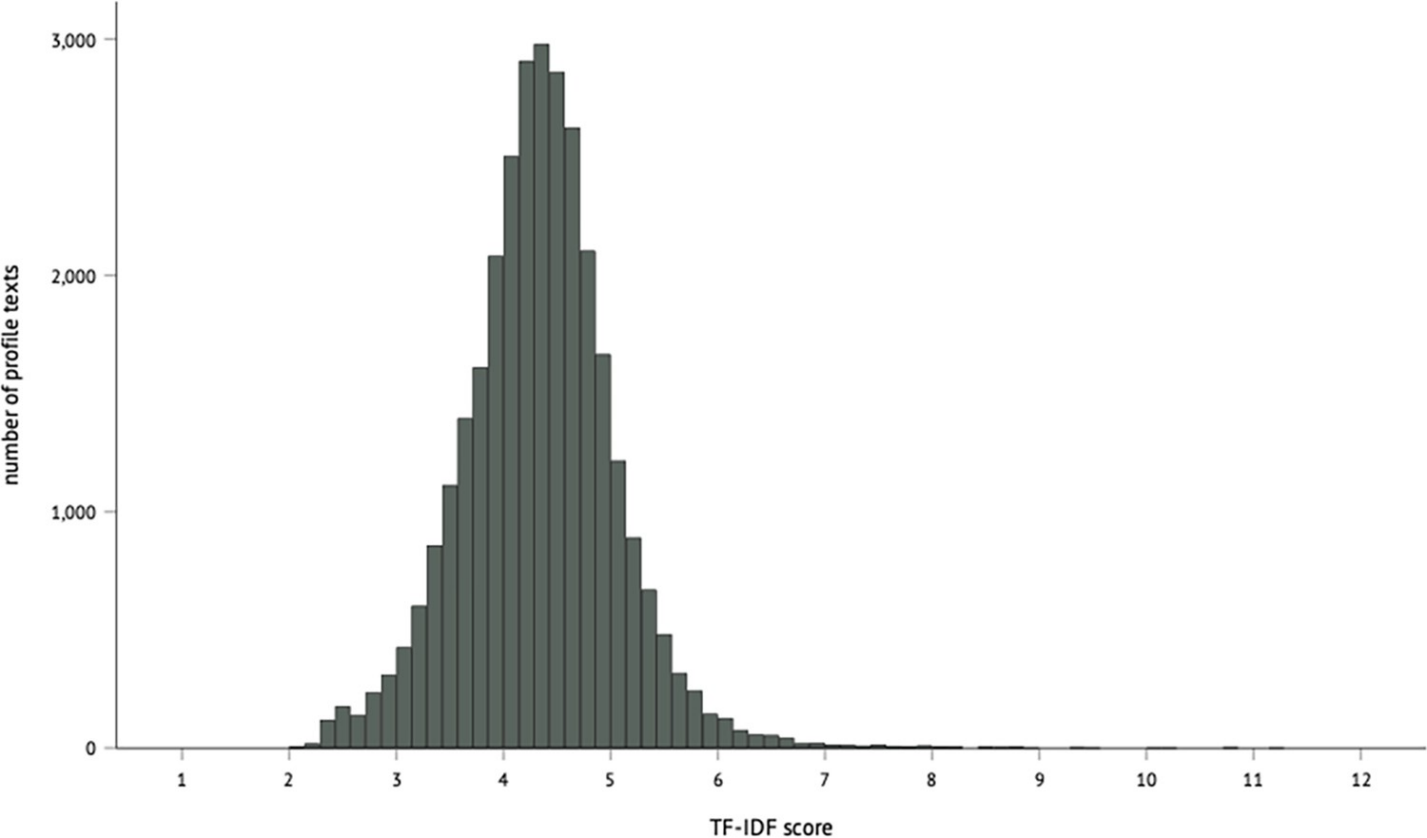
Distribution of the TF-IDF scores of all 31,163 profile texts in the sample.

The TF-IDF score distribution corroborated the initial impression that only few texts were original in their word use, which is illustrated in Fig 2. All 31,163 texts were therefore divided into seven bins, based on the percentiles of the TF-IDF score. This was done for both texts that were written by people who indicated to be men (*n* = 17,869) and for those who indicated to be women (*n* = 13,294), as participants in the perception study saw profiles written by people of their sexual preference. The seventh bin–containing the texts with the highest TF-IDF scores–contained all texts falling in the range until the 40% percentile of TF-IDF scores. Each of the other bins contained all texts within the next 10^th^ percentile. To illustrate this for the texts written by men: the highest TF-IDF score was 11.19 and the lowest score 2.15, which means that for texts of men the TF-IDF scores in a bin differed 0.90 (11.19–2.15/10). As such, all texts that scored between 2.15 and 3.06 were part of the first bin (the lowest score plus 0.90), and those scoring between 3.06 and 3.96 were part of the second bin (3.05 plus 0.90), and so on. Table 1 below provides for the profiles in each of the bins the lowest and highest TF-IDF score, the percentile score, and the number of profiles included.

**Table 1:** Distribution of texts written by men and women in seven bins based on percentiles of the TF-IDF scores.

		Profile texts written by men			Profile texts written by women		
Bin	Percentile	Lowest TF-IDF score	Highest TF-IDF score	*n*	Lowest TF-IDF score	Highest TF- IDF score	*n*
1	0 to 10th	2.151	3.055	665	2.053	2.789	213
2	10th to 20th	3.055	3.958	4,125	2.789	3.525	1,271
3	20th to 30th	3.958	4.862	9,341	3.525	4.260	4,657
4	30th to 40th	4.862	5.765	3,225	4.260	4.996	5,479
5	40th to 50th	5.765	6.669	409	4.996	5.732	1,400
6	50th to 60th	6.669	7.572	72	5.732	6.468	203
7	60th to 100st	7.572	11.186	665	6.468	9.412	71
Total				17,869			13,294

*Note*. *n* indicates the number of profile texts included in the bin in that percentile.

To end up with a total of approximately 300 profile texts, 22 texts were randomly selected from each of the seven bins, resulting in a total of 154 texts written by men and 154 by women, that is, 308 texts altogether.

All texts were accompanied by a different blurred profile picture, which was a picture of a person with the same sex as the text’s writer. The texts and pictures were then combined into one dating profile. The layout of the profiles is exemplified in Fig 1. Since the texts we used for our materials included parts of authentic profile texts, the profiles that we have used in this study are only available upon request.

#### Procedure

The perception study was conducted online and took approximately ten minutes to complete. First, participants were welcomed and informed about the procedure of the experiment. After participants provided written informed consent in the online survey itself, the experiment started by answering a few demographic questions (e.g., gender, age). Based on their indicated sexual preference, participants were then presented with five profiles that were randomly chosen from the selection of 154 profile texts written by men or women. Since participants were informed that they could end the experiment at any time, not all participants evaluated five profiles. Participants saw one profile at a time; after each profile, participants assessed the originality of the profile and were asked about perceptions of the personality (intelligence, sense of humor, oddness) and attractiveness (physical, social, romantic attractiveness) of the profile owner. Before the next profile was presented, participants answered with a yes/no question whether they would like to go on a date with the profile owner. Finally, the participants who ran through the entire experiment were thanked, debriefed, and allowed to leave comments, and were then redirected to the contact form of the dating site which they could fill in if they had interest in participating in a raffle for a three-months free membership. This raffle was carried out by the dating sites.

#### Measures

Except for intention to date, all variables in this study were measured on a Likert scale from 1 (*completely disagree*) to 7 (*completely agree*). Intention to date was measured using one binary yes/no question: “I would like to go on a date with this person”. The study’s independent variable was perceived profile text originality, which was measured with the item “This profile text seems original to me”. All other items were predominantly derived from existing scales, with the wording translated and slightly adjusted to fit our experiment.

Each of the three mediation variables of this study was measured by two items. Perceived intelligence was measured with the items “I think this person is smart/intelligent” (based on [55]; Pearson’s *r* = .83), perceived sense of humor with the items “I think this person has humor/is funny” (following [56]; Pearson’s *r* = .82), and perceived oddness was measured with the items “I think this person is odd/peculiar” (following [39,40]; Pearson’s *r* = .77).

The outcome variables of attractiveness were measured with three items, each covering another dimension of perceived attractiveness: physical attractiveness (“I think this person is good-looking”), social attractiveness (“I think this person is kind”; [57]), and romantic attractiveness (“I could fall for this person”; [58]).

#### Statistical analysis

All participants who rated at least one dating profile from the full set of 308 profiles were included in the dataset, with a maximum of five profiles (*M* = 2.95, *SD* = 1.50). In total, 775 participants (62.8%) viewed and assessed the maximum of five profiles. Seven participants who did not want to indicate their sexual preference, were presented with a total of ten profiles of which five were from men and five from women. This together resulted in 4289 individual profile assessments. The 308 texts differed in the number of times they were rated, ranging between 7 and 20 ratings per text (*M* = 14.10, *SD* = 2.56). With the irrNA package in R [59], intraclass correlation coefficients (ICC; 1,*k*) were calculated for all eight impression formation variables, providing an indication of the internal consistency of the scores given to the different texts by the different participants. All ICC’s were between .66 and .85 (ICC_mean_ = .80; see file ICC Scores on OSF for further details), indicating a good reliability between scores given by participants [60]. Consequently, data was aggregated on text level and mean scores were calculated for each of the variables. For dating intention, which was measured with a dichotomous yes/no question, the text mean score ranged between 0 and 1, with higher scores indicating more willingness to date the profile owner.

To test the mediation hypotheses, we used model 4 of the PROCESS v3.1 macro in SPSS [61] with a bootstrapping approach with 10,000 samples and 95% Monte Carlo confidence intervals. The independent variable was the perceived profile text originality score. Perceived physical, social and romantic attractiveness and dating intention were the dependent variables, and perceived intelligence, sense of humor, and oddness the mediating variables. The data underlying this article are available on OSF, at: https://osf.io/yns83/.

### Results

Before conducting mediation analyses, a multivariate regression model revealed that perceived text originality significantly predicted all seven mediating and outcome variables, *F*(7, 300) = 87.41, *p* < .001, η_p_^2^ = .671. Table 2 provides all mean scores, standard deviations, and correlation scores of perceived text originality scores and the mediating and outcomes variables. To check on potential differences in the assessments between the members of 50PlusMatch and Pepper, we also run analyses of the 50PlusMatch and Pepper participants separately. As similar results were found, the results reported are those with the participant group as a whole. Moreover, results of the mediation analyses were similar when word count was included as a control variable, hence the results without word count as control variable are presented.

**Table 2:** Means, standard deviations, and correlations among perceived text originality scores and all impression formation variables.

Variable	Mean (SD)	1	2	3	4	5	6	7
1. Text originality	3.69 (0.91)							
2. Intelligence	3.93 (0.69)	.74*						
3. Humor	3.83 (0.56)	.73*	.64*					
4. Oddness	3.59 (0.64)	-.30*	-.33*	-.31*				
5. Physical attractiveness	3.79 (0.41)	.51*	.61*	.59*	-.30*			
6. Social attractiveness	4.25 (0.51)	.60*	.61*	.68*	-.59*	.63*		
7. Romantic attractiveness	3.32 (0.82)	61*	.66*	.65*	-.61*	.63*	.77 *	
8. Intention to date	0.30 (0.20)	.60*	.62*	.58*	-.52*	.56*	.67*	.83*

*Note*. * *p* < .01. All variables were measured on a seven-point Likert scale, except for ‘intention to date’ which was measured using a dichotomous yes (1)/no (0) question.

Hypothesis 1 proposed that perceived profile text originality increases perceptions of profile owners’ intelligence which, in turn, positively affect perceptions of profile owners’ attractiveness and dating intention. Results indicated that perceived text originality was indeed a significant predictor of perceived intelligence: owners of profile texts that scored higher on originality also received higher scores on perceived intelligence, *b* = 0.56, *SE* = 0.03, *p* < .001, CI: 0.50, 0.61. Perceived intelligence was a significant predictor of physical, *b* = 0.24, *SE* = 0.04, *p* < .001, CI: 0.16, 0.32, social, *b* = 0.13, *SE* = 0.04, *p* = .001, CI: 0.05, 0.20, and romantic attractiveness, *b* = 0.35, *SE* = 0.06, *p* < .001, CI: 0.23, 0.47. In addition, participants were more willing to date profile owners they perceived to be intelligent, *b* = 0.08, *SE* = 0.02, *p* < .001, CI: 0.04, 011. The data thus confirm H1.

The second hypothesis stated that perceived profile text originality positively affects attractiveness perceptions and dating intention through higher humor perceptions. As hypothesized, higher scores on perceived profile text originality significantly predicted higher scores on humor perceptions, *b* = 0.45, *SE* = 0.02, *p* < .001, CI: 0.40, 0.50. Perceived humor was, in turn, a significant predictor of physical, *b* = 0.27, *SE* = 0.05, *p* < .001, CI: 0.18, 0.37, social, *b* = 0.38, *SE* = 0.05, *p* < .001, CI: 0.29, 0.47, and romantic attractiveness, *b* = 0.46, *SE* = 0.07, *p* < .001, CI: 0.31, 0.60, as well as of dating intentions, *b* = 0.06, *SE* = 0.02, *p* = .003, CI: 0.02, 0.10. H2 is thus supported.

Hypothesis 3 posed that perceived profile text originality negatively impacts attractiveness and dating intentions through increased oddness perceptions. In contrast with H3, higher profile text originality scores led to lower scores on perceptions of profile owners’ oddness, *b* = -0.21, *SE* = 0.04, *p* < .001, CI: -0.28, -0.13. Higher perceptions of oddness did have a negative effect on perceptions of social attractiveness, *b* = -0.31, *SE* = 0.03, *p* < .001, CI: -0.37, -0.26, romantic attractiveness, *b* = -0.52, *SE* = 0.05, *p* < .001, CI: -0.61, -0.43, and dating intention, *b* = -0.10, *SE* = 0.01, *p* < .001, CI: -0.08, -0.13, but not on physical attractiveness, *b* = -0.05, *SE* = 0.03, *p* = .087, CI: -0.11, 0.01. H3 is thus not supported by the data: higher scores on perceived oddness negatively affect perceived attractiveness and dating intention, but profile owners whose text scored higher on text originality scored lower on oddness. Fig 2 shows the results of the mediation analyses for all four outcome variables.

**Fig 3:**
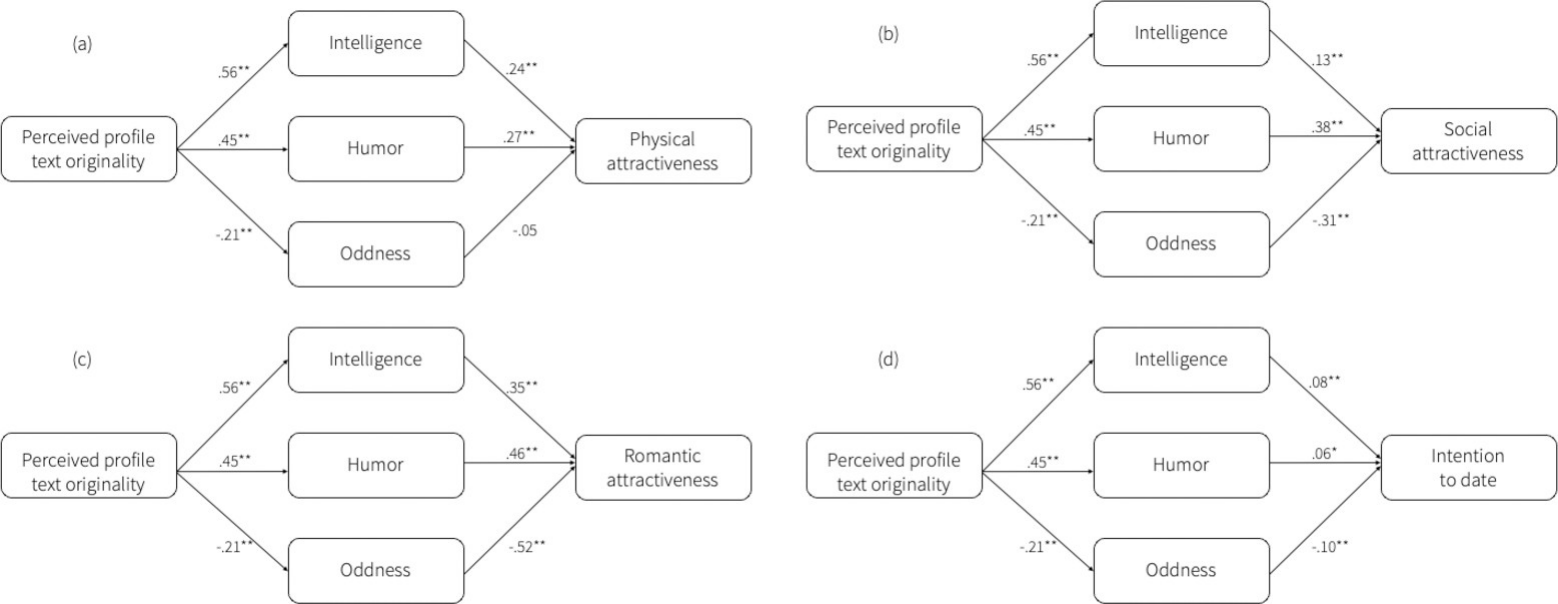
Results of the four mediation analyses displaying that the effect of perceived profile text originality on (a) physical attractiveness is mediated by perceived intelligence and humor, and that the effect of profile text originality is mediated by perceived intelligence, humor, and oddness for (b) social attractiveness, (c) romantic attractiveness, and (d) dating intention, but for oddness in the opposite direction than hypothesized. The coefficients represent the unstandardized coefficients. *Note*. ** *p* < .001, * p < .01. All variables were measured on a seven-point Likert scale, except for ‘intention to date’ which was measured using a dichotomous yes (1)/no (0) question.

After controlling for the mediators, perceived text originality is no longer a significant predictor of all three attractiveness dimensions, indicating full mediation (physical: *b* = -0.04, *SE* = 0.03, *p* = .268, CI: -0.10, 0.03, social: *b* = 0.03, *SE* = 0.03, *p* = .385, CI: -0.04, 0.09, romantic: *b* = 0.03, *SE* = 0.05, *p* = .500, CI: -0.07, 0.13). This indicates that perceived profile text originality only affects the attractiveness variables through the three mediating variables. The mediators partially mediate the effect of perceived profile text originality on dating intention, as the direct effect is still significant after controlling for the mediators, *b* = 0.04, *SE* = 0.01, *p* = .007, CI: 0.01, 0.07. Thus, text originality affects dating intentions through increased perceptions of intelligence and sense of humor and decreased perceptions of oddness. However, originality also directly affects dating intentions, implying that other factors might further explain this relationship Fig 3.

### Discussion

The perception study confirmed our hypothesis that perceived profile text originality affects impression formation. Owners of profiles scoring higher on text originality were evaluated as more intelligent and humorous and, in turn, as more attractive, supporting H1 and H2. However, in contrast to our expectations, higher text originality scores negatively affected perceptions of profile owner’s oddness. H3 was not supported: Higher scores on perceived oddness had a negative impact on attractiveness, but owners of more original profiles were not seen as more, but as less, odd.

These data showed that, overall, perceptions of profile text originality positively affect impressions of the profile owner’s personality and attractiveness. However, these results do not show which specific features of the profile text affect perceptions of originality. Therefore, we now turn to a content analysis to identify which profile text characteristics increase perceptions of originality.

## Content analysis: Textual characteristics of profiles perceived as original

### Method

In this section, we describe the two stages of the coding procedure that were undertaken in this exploratory analysis to answer the research question on the textual characteristics that predict the perceived originality of online dating profile texts. This section first reports on the qualitative analyses we did to compile a codebook consisting of several main categories that were captured by different features, followed by a more detailed description of the content analysis stage in which all 308 profiles were coded on the 15 features of the codebook. The entire codebook can be found in the Online Supplementary Materials and on OSF.

#### Qualitative analysis

In the first stage of the coding procedure, we conducted a qualitative analysis of a subselection of 60 of the 308 profile texts, differing in received originality scores: the 20 texts that scored highest on perceived profile text originality (range: 5.69–5.00; *M* = 5.29, *SD* = 0.21), those 20 that scored lowest (range: 2.27–1.25; *M* = 1.88, *SD* = 0.30), and the 20 texts that scored closest to the mean originality score of 3.69 (range: 3.75–3.63, *SD* = 0.91).

These 60 texts were all coded on several features of the three main categories of text characteristics that we distinguished in the codebook: stylistic features, self-disclosure, and perspective-taking. To develop the codebook, we relied on bottom-up and top-down coding. This means that the main categories and features we developed were both driven by literature and the data. The form-related features, such as the use of metaphors and concrete language as indicators of expressive and vivid language use, were in particular prompted by prior literature (e.g., [28,50,51]). The data-driven features were identified based on patterns and themes among texts within a group, or differences between the three groups of texts. These features seemed to be more concerned with the profiles’ content, for which we could not rely on existing literature about dating profile originality, as literature is scarce on this topic. Examples of such features in the codebook are the number of self-disclosure statements (main category: self-disclosure) or the perspective from which the profile text was written (main category: profile perspective). The resulting three main categories and features included in the final codebook are explained in more detail below.

First, texts that scored higher on perceived originality more often seemed to contain stylistic characteristics such as vivid descriptions and imagery than those who scored lower on perceived originality. As opposed to those less original texts, original texts seem to be more likely to contain metaphorical expressions (e.g., “I am a very good cook” vs. “I am a star in the kitchen”), and particularly novel metaphors (e.g., “I don’t like growers of crops situated between nose and neck”). In addition, original profile texts seem to include more low-frequent words, adjectives, and adverbs than less original texts, which are other features that can evoke imagery. These five stylistic features are thus included in the codebook as features potentially predicting perceived profile text originality.

Second, the qualitative analysis suggested that texts scoring higher on perceived originality contain more self-disclosure statements than texts scoring lower on perceived originality. In our preliminary analysis, we saw that self-disclosure was presented in several ways. First, original texts seemed to contain more self-disclosure (e.g., “I’m a 50-year old man” vs. “I’m a loyal 50-year old man with a high sense of humor”) and more intimate self-disclosure statements (e.g., “I often go to the gym” vs. “In my life, sharing is the keyword”). Moreover, self-disclosure statements in profiles deemed more original also appeared to be more concrete compared to less original profiles, which means revealing personal information that activates detailed (image-based) representations of objects or events [62] (e.g., “Food is essential for me” vs. “Coffee and a cracker with cheese or jam are essential in my morning ritual”). As additional features of self-disclosure, we decided to take into account the number of words and the percentage of I-references, which are also associated with self-disclosure [63,64], as well as article use, which is seen as another measure of language concreteness [65]. A total of six self-disclosure features is thus added to the codebook.

Third, profiles scoring high on perceived originality seemed to be less self- and more other-focused. First, some original texts were partially or completely written from another perspective: these profile owners also used another person’s perspective or another person’s quotes to present themselves (see Fig 1A and 1C for an example). Second, we noticed that original texts seemed to contain more information about the kind of relationship (partner) the profile owner was looking for (e.g., “Looking for an intelligent man with a sense of humor”). Moreover, we took into account the percentage of question marks and you-references as these can be indicators of the profile writer’s attention being directed to the potential partner and not just the self [64]. These four perspective-taking features are therefore included in the codebook.

#### Content analysis

In the second stage, all 308 texts, including the 60 texts analyzed in the initial phase, were coded on all features defined in the codebook, of which seven were coded manually and eight automatically. For the manually-coded features, two judges who were blind to the profile text originality score coded a random subset of texts (*n* = 30). We used the Kappa statistic to calculate the intercoder reliability and used the benchmark of Landis and Koch [66] to determine strength of agreement. One coder then went on to individually code the remaining profiles. Manual coding happened on two levels: profile perspective was coded on the level of the profile text, but since multiple stylistic and self-disclosure features could occur within a text–and even a single sentence–these features were coded at clause level. A clause was identified as the smallest unit in a sentence that was interpretable on its own. Parts of enumerations and elliptical expressions (e.g., “philosopher, adventurous”, “no standard man”) were considered as separate clauses. Profile texts consisted on average of 7.3 clauses (*SD* = 5.9). Clauses that were (part of) quotations of others (*n* = 16 texts with (only) citations) were not coded on stylistic and self-disclosure features (e.g., John Weir’s quote “Love myself I do. Not everything, but I love the good as well as the bad”). For automatic coding, we used the Linguistic Inquiry and Word Count program (LIWC) [67] and T-Scan, a software tool for analyzing Dutch texts [68].

*Stylistic features*. Of the five stylistic features, two were manually coded: presence of (1) fixed (e.g., “I have my life back on track”) and (2) novel metaphors (e.g., “Looking for a Don Juan who can make me weak at the knees”) (κ’s of .80 and .87, respectively). Fixed and novel metaphors were included as a binary variable (0 = absent, 1 = present) because the relatively short profiles resulted in little variation in the number of occurrences. Proportions of adjectives, adverbs, and low-frequent words were automatically coded, with low-frequent words defined as the proportion of words not part of the 20,000 most frequent words of the Dutch, derived from the SoNaR corpus [69]. Because the TF-IDF score signals variation in the words used in a text compared to the other texts, TF-IDF score was also taken into account as a stylistic feature.

*Self-disclosure features*. Three of the six self-disclosure features were coded manually. Each clause in which a profile owner reveals personal information was coded as a self-disclosing clause, and each self-disclosure clause was manually coded on three features. First, within one clause, the total number of self-disclosing expressions were counted, which involved only information about the profile owner him- or herself (e.g., underlined words indicate the four self-disclosing statements in “I’m a loyal 50-year old man with a high sense of humor”) and not the type of relationship (partner) the profile owner was looking for (e.g., “My future partner should be funny and smart”).

Second, the intimacy of the self-disclosed information was coded (κ = .76). To do this, we followed the classification scheme of Altman and Taylor [70] adapted by Sharabi and colleagues [71] for dating contexts. Each self-disclosure clause was coded as containing either biographical (low intimate; e.g., name, age), evaluative (medium intimate; e.g., personality traits, hobbies), or normative/moral personal information (high intimate; e.g., norms and values, confessions). All self-disclosing statements and intimacy scores (1, 2, 3) in self-disclosing clauses of a text were summed to calculate the total number of self-disclosing statements and the overall intimacy score of a text.

The third feature within this category was the concreteness of self-disclosed information (κ = .81). To be identified as concrete, information in a profile should evoke clear imagery. For instance, by describing an assignable location (e.g., “A road trip across Australia is on my bucket list”), product (e.g., “My favorite book is Harry Potter”), activity (“I prefer mountain biking to Nordic walking”), period or moment (e.g., “I got divorced in 1999”). This could also involve information that specifies information in other (generic) self-disclosing clauses, such as the second clause on sushi in “I love cooking. Home-made sushi is my specialty”, which further specifies the information disclosed about the profile owner’s love to cook. Concreteness was eventually included as a binary variable (absent or present) because of the low number of occurrences within texts.

The remaining three features related to self-disclosure were coded using LIWC, which were the number of words, and the percentage of I-references and articles in the profiles.

*Perspective-taking features*. First, profile perspective was coded on profile text level. Self-perspective profiles were those profiles that were (fully) written from the profile owner’s own perspective (e.g., “I am a man who hopes to find a friendly woman”; see also Fig 1C and 1D), while profiles that were not (fully) written from the self- but also from another perspective were coded as no self-perspective (see also Fig 1A and 1B; all κ’s > .63). For instance, profiles that were written from a 3^rd^ person perspective (“This man hopes to find a friendly woman”). Second, we coded the number of clauses that included information about the kind of relationship (partner) a profile owner seeks (κ = .87), but given the low frequency in which this occurred, we eventually included this feature as a binary variable (absent or present). The percentage of question marks and you-references were automatically calculated using LIWC.

### Results

All mean scores, standard deviations, and correlation scores of text originality scores and all 15 included features are provided in Table 3. We found that profiles were more likely to include (at least) one fixed metaphor (*n* = 132; 42.9%) than a novel metaphor (*n* = 95; 30.8%). The percentage of low-frequent words (85.4%, indicating the high percentage of high-frequent words), adjectives (12.2%), and adverbs (6.02%) in the profiles was relatively low. Results of the self-disclosure features showed that profiles included 53.9 words on average, in which profile owners expressed on average 9.17 self-disclosing statements. In somewhat more than half of the profiles, concrete self-disclosing information was provided (*n* = 161; 52.3%). In addition, the percentage of articles and I-references in the profiles was 6.88 and 5.81, respectively. A large majority of profiles were fully written from the profile owner’s own perspective (*n* = 242; 78.6%), and in almost half of the profiles no information was revealed about the type of relationship (partner) the profile owner sought for (*n* = 153; 49.7%). On average, the percentages of you-references and question marks were 1.35 and 0.48.

**Table 3:** Means, standard deviations, and correlations among text originality scores and all coded features.

Category	Feature	Mean (SD)	1	2	3	4	5	6	7	8	9	10	11	12	13	14	15
	1. perceived text originality	4.44 (0.91)															
Stylistic features	2. TF-IDF	5.05 (1.78)	.21**														
	3. fixed metaphors^a^	42.9 (49.6)	.24**	-.07													
	4. novel metaphors^2^	30.8 (46.2)	.29**	.18**	.06												
	5. low-frequent words^b^	85.4 (18.4)	.01	-.77**	.13*	-.03											
	6. adjectives^b^	12.2 (9.16)	.11	-.03	.20**	.09	.08										
	7. adverbs^b^	6.02 (5.11)	.20**	-.06	.11	.11	.28**	-.01									
Self-disclosure features	8. number of words	53.9 (26.8)	.56**	.37**	.19**	.28**	-.01	-.09	.31**								
	9. number of self-disclosures	9.17 (8.16)	.45**	.27**	.19**	.16**	-.07	.31**	.13*	.47**							
	10. concrete self-disclosure^a^	52.3 (50.0)	.42**	.21**	.09	.13*	-.07	-.03	.15**	.46**	.48**						
	11. article use^c^	6.88 (4.95)	.04	-.37**	-.05	.08	.47**	-.20**	-.01	-.04	-.21**	-.06					
	12. I-references^c^	5.81 (4.28)	-.11	-.49**	-.03	-.17*	.46**	-.13*	-.02	.01	-.10	-.10	.04				
Perspective-taking features	13. no self-perspective^a^	21.0 (41.1)	.03	.48**	-.09	-.04	-.42**	.16**	-.08	-.10	-.03	.01	-.20**	-.50**			
	14. looking-for clauses^a^	50.3 (50.1)	.09	-.34**	-.01	-.03	.17**	-.03	-.08	.13*	-.33**	-.16**	.15*	.05	-.10		
	15. you-references^b^	1.35 (2.13)	-.02	-.16**	-.08	-.02	-.18**	-.10	-.08	-.06	-.13*	-.13*	-.12*	.23**	-.12*	-.07	
	16. question marks^b^	0.48 (1.38)	.01	-.01	.10	-.06	-.01	-.09	.03	-.04	-.08	.02	.01	.01	.00	-.001	.10

*Note*. ^a^ Features measured on a binary scale (0 = absence of the feature; 1 = presence of the feature). ^b, c^ Features presenting proportion (^b^) or percentage (^c^) scores: Number of words in that category divided by total number of words in text (for percentage scores multiplied by 100).

^a, b, c^ are all presented as percentages scores in the mean and SD column.

* indicates *p* < .05 and

** *p* < .01.

#### Regression analysis

The hierarchical multiple regression analysis that was performed next included all coded features as predictor variables. The dependent variable was the perceived originality score assigned to all 308 texts by participants in the perception study. Table 4 presents the results of this regression analysis. As the number of self-disclosures and self-disclosure intimacy were highly correlated (.83), this led to multicollinearity issues in our analysis. Therefore, only the total number of self-disclosure statements was included. The stylistic features were entered in the regression first, *F*(6, 301) = 13.78, *p* < .001, *R*^2^ = .215. Fixed metaphors, novel metaphors, TF-IDF, and low-frequent words all made a significant contribution to this first model (with all *β’s* ≥ .20 and *≤* .41, *p’s ≤* .002) and these stylistic features accounted for 21.5% of the variation in originality scores. The self-disclosure features explained an additional 20.9% of the variance, *F*(5, 296) = 21.52, *p* < .001, *R*^2^ = .209. Except for the use of I-references, all self-disclosure features contributed significantly to the model (with all *β’s* ≥ .13 and *≤* .41, *p’s ≤* .023). Finally, the addition of the perspective-taking features did not add significantly to the model, *F*(4, 292) = 1.80, *p* = .128, *R*^2^ = .014.

**Table 4:** Summary of Hierarchical regression analysis for text features predicting perceived profile text originality.

		Model 1			Model 2			Model 3		
Category	Features	B	*SE B*	β	B	*SE B*	β	B	*SE B*	β
Stylistic features	TF-IDF	0.21	0.04	.41***	-0.05	0.05	-.11	-0.08	0.05	-.16
	fixed metaphors^a^	0.36	0.10	.20***	0.18	0.09	.10*	0.19	0.09	.10*
	novel metaphors^a^	0.38	0.10	.20***	0.20	0.09	.10*	0.23	0.09	.12*
	low-frequent words^b^	1.36	0.43	.28**	-0.52	0.45	-.11*	-0.66	0.46	-.13
	adjectives^b^	0.43	0.52	.04	1.04	0.53	.10	0.89	0.54	.09
	adverbs^b^	1.92	0.99	.11	0.59	0.91	.03	0.64	0.91	.04
Self-disclosure features	number of words				0.01	0.00	.41***	0.01	0.00	.42***
	number of self-disclosures				0.02	0.01	.15*	0.02	0.01	.19**
	concrete self-disclosure^a^				0.30	0.10	.17**	0.31	0.10	.17**
	article^c^				0.02	0.01	.13*	0.03	0.01	.16**
	I-references^c^				-0.01	0.01	-.06	.000	0.01	-.01
Perspective-taking features	no self-perspective^a^							0.29	0.13	.13*
	looking-for clauses^a^							0.04	0.09	-.02
	you-references^c^							0.02	0.02	.06
	question marks^c^							0.02	0.03	.03
*R* ^2^				.215			.425			.438
Adjusted *R*^2^				.200			.403			.410
*ΔR* ^*2*^				.215***			.209***			.014

*Note*. ^a^ Features measured on a binary scale (0 = absence of the feature; 1 = presence of the feature). ^b, c^ Features presenting proportion

(^b^) or percentage

(^c^) scores: Number of words in that category divided by total number of words in text (for percentage scores multiplied by 100).

* indicates *p ≤* .05

** *p* < .01

*** *p* < .001.

As shown in Table 4, the final model with all 308 profiles and all features included reveals seven features significantly predicted perceived profile text originality; number of words was the strongest predictor, followed by the number of self-disclosing statements, the presence of concrete self-disclosure, the use of articles, and then the presence of: no self-perspective, novel metaphors, and fixed metaphors. Together the features explained 43.8% of the variance in text originality scores.

Moreover, we have run a similar regression without the results of the 60 profiles that were used in the qualitative analysis phase to construct the codebook. The results of the regression with only the 248 profiles are comparable: adding stylistic features first accounted for 19.3% of the variance in originality, *F*(6, 241) = 9.59, *p* < .001, *R*^2^ = .193, followed by the self-disclosure features that explained an additional 17.3%, *F*(5, 236) = 12.89, *p* < .001, *R*^2^ = .173. Similar to the results with all profiles included, the perspective-taking features did not contribute significantly to the model, *F*(4, 232) = 1.18, *p* = .321, *R*^2^ = .013.

### Discussion

The goal of the content analysis was to gain insights into what profile text characteristics could foster positive perceptions of profile text originality. To do so, all texts were coded on a number of textual features that were determined based on an initial content analysis and could be predictors of perceived profile text originality. Each feature fits one of the three text feature categories we distinguished, that is, stylistic features, self-disclosure statements, or perspective-taking features. We then ran regression analyses to identify which features predict perceptions of text originality, as scored by the participants in the perception study.

Our results reveal that primarily the stylistic and self-disclosure features were correlated positively with perceived text originality scores and explained most of the variance in originality scores. With regard to stylistic features, our findings show that profiles that score higher on perceived profile text originality are more likely to contain one or more fixed or novel metaphors. Considering self-disclosure, we found that both features that looked at the quantity (i.e., total number of words and total number of self-disclosing statements) as well as quality of the self-disclosure (i.e., the occurrence of concrete self-disclosure and article use) predicted text originality scores. Although the profile perspective was found to be a significant predictor of text originality, the perspective-taking features did overall not contribute to the model.

## General discussion

As far as we are aware, this is the first study that has focused on perceived originality in online dating profiles. In the perception study, we first investigated the effects of perceived profile text originality on impression formation. This was done by presenting actual users of web-based dating sites with dating profiles which they evaluated on the profile’s originality and the profile owner’s personality and attractiveness. Next, we conducted a content analysis to explore what characteristics in a dating profile text increase perceptions of profile text originality.

Results of the perception study show that higher scores on perceived intelligence and sense of humor mediate the positive relationship between perceived profile text originality and impressions of attractiveness and dating intention (H1 and H2). This positive correlation of perceived originality, intelligence, sense of humor, and attractiveness accords with correlations found in prior studies [26–28]. Contrary to the expectations in H3, we found that higher originality scores lead to lower rather than higher oddness scores. In line with our expectation, profile owners scoring higher on perceived oddness scored lower on attractiveness and dating intention.

The perception study data showed thus that, overall, perceptions of profile text originality positively affect impressions of the profile owner’s personality and attractiveness, but the content analysis provides insights into what profile text characteristics could increase these text originality perceptions. Our results reveal that primarily stylistic and self-disclosure features predicted higher text originality scores. It seems that profiles that were perceived as more original were more likely to contain fixed and novel metaphors (stylistic features), and more and concrete self-disclosures (self-disclosure features). Finally, profiles deemed original were less likely to be (fully) written from a self-perspective (perspective-taking feature).

### Implications and directions for future research

This study yields several implications for theory and future studies on (the effects of) originality. First, our study reveals that a general consensus exists among the online dating site users of this study about what profile texts are original and not. Moreover, the participants showed high agreement on the owners of which profiles were considered odd, and these profiles scored low on originality. Consistent with the two-dimensional concept of creativity [12], this finding suggests that, without being instructed to do so, online daters apply novelty and appropriateness criteria to assess a profile’s originality; only profiles that are both novel *and* appropriate are considered original, profiles that are just novel are not. This raises the question where to draw the line between profiles that are novel but not appropriate, profiles that are appropriate but not novel, and profiles that are both novel and appropriate. A future study could investigate this by asking participants to evaluate the perceived novelty and appropriateness of a large set of texts instead of the text’s overall perceived originality.

Second, the results of the perception study show that online daters use profile originality as a cue to form impressions about profile owners. More specifically, it seems that a profile’s originality primarily leads to positive impressions, both with regard to perceptions about the profile owner’s personality (higher scores on intelligence and sense of humor), and the profile owner’s attractiveness and participants’ dating intentions. This positive effect of originality on impression formation is further corroborated by the finding that perceived originality did not lead to higher scores on perceptions of the less desired trait oddness. Originality may thus be seen as a positive characteristic of a dating profile, which accords with previous interview studies in which online daters expressed negative attitudes towards dating profiles lacking originality [16,17]. However, as the participants of the present study were older adults who are members of dating platforms on which the textual component on a dating profile plays a prominent role, these results need to be corroborated among younger samples as younger adults are often more inclined to use dating applications with more picture-based dating profiles. It would be interesting to investigate how different dating demographics define and appreciate originality in dating profile texts.

Third, the results of the exploratory content analysis suggest that originality is a multifaceted construct in online dating: perceptions of text originality are affected by choices of form (stylistic features) as well as meaning (self-disclosure statements). This suggests that in addition to a multidimensional construct (i.e., novel and appropriate), originality is manifested through both meaning and form characteristics in dating profiles. Future research should examine how the criteria of novelty and appropriateness on the one hand, and meaning and form on the other hand, relate to each other. For example, stylistic features may be form characteristics that can boost a profile’s novelty, while self-disclosure features may be meaning characteristics that are added to satisfy appropriateness criteria. The latter assumption builds upon an earlier study that suggested that online daters reveal personal information to conform with contextual expectations [24].

Our findings may well extend to other text genres, such as job application letters or consumer-to-consumer advertisements. There, text originality may also be a balancing act between novelty and appropriateness. Moreover, it is also likely that in these and other texts, originality is not only defined by form, but also by certain meaning characteristics that are specific to the context. For example, a consumer-to-consumer advertisement should not only be original in form, but should perhaps also always contain specific product information in order to be perceived as original. Whether these assumptions hold in other contexts though, is up for future studies.

Fourth, this study has shown that it is possible to assess perceived text originality from authentic profile texts based on content analytical features. Our methodological approach offers opportunities for other research aiming to investigate what constitutes originality in texts and how perceived originality affects evaluations. With the features coded in this study, we were able to explain nearly half of the variation in perceived profile text originality scores, and particularly the manually-coded features were important in this. A next challenge would be to examine whether automated measures of the manual-coded features of this study that seemed to indicate perceived text originality, could be developed using natural language processing (NLP) techniques, such as feature extraction and language modeling.

The use of authentic online dating profile texts is thus one of the study’s strengths. At the same time, ethical issues can and should be taken into consideration when using authentic texts. When conducting this study, we had obtained ethical approval of our local REDC, and we made every effort to ensure that sentences and phrases used in our stimuli could not be traced back to the original writers. Nevertheless, a debate has emerged in social sciences recently (e.g., [72]): can people’s online texts be used for scientific analyses, even when these texts are publicly available, if the writers of those texts are not aware of this? There is no simple answer to this, and much depends on the specific online platform and the exact purpose of the study. This is an important consideration for future studies looking into communicative practices in online communicative settings, ranging from BTL reader comments on news sites to online dating.

## Conclusion

To conclude, the results of this study accord well with the recommendations of dating sites to write original profile texts: the originality of an online dating profile text is indeed used as a cue for impression formation which has primarily positive effects. Owners of profiles that score high on profile text originality tend to score higher on other positive dimensions, that is, intelligence, sense of humor, and attractiveness, as well as on participants’ intentions to date the profile owner. Given our results, there seem to be (at least) two ways to increase perceptions of profile text originality: by disclosing more and concrete personal information, and by using stylistic features, such as (fixed and novel) metaphors. Altogether, our study highlights the importance of perceived originality in online dating profile texts, which can be manifested through both *what* information is provided and *how* this information is presented.

## Supporting information

S1 File (DOCX)
