# Supplementary material for: Originality in online dating profile texts: How does perceived originality affect impression formation and what makes a text original?
Source: PLoS One. 2022 Oct 19;17(10):e0274860. doi: 10.1371/journal.pone.0274860 (PMC9581348; doi:10.1371/journal.pone.0274860)
Supplement: S1 File — (DOCX) [file pone.0274860.s001.docx]

| CODEBOOK OF FEATURES | | | | |
| --- | --- | --- | --- | --- |
| Category | Feature | Definition | Measured/measurement level | Examples |
| Stylistic features | Presence of fixed metaphors | The occurrence of a metaphorical expression in the profile text that is (a) a fixed word combination that is used metaphorically, or (b) a trope, meaning a metaphorical expression that is particularly common in the context of relationships and (online) dating. | Manual coding;  Binary: absent (0) or present (1) in the text | “having a warm personality” (fixed word combination)  “I’m a glass half full type” (fixed word combination)  “building a relationship” (trope)  “see whether there is chemistry” (trope) |
|  | Presence of novel metaphors | The occurrence of a metaphorical expression in the profile text that appears novel and is not a fixed word combination that is used frequently or a trope that is often heard in a dating context. | Manual coding;  Binary: absent (0) or present (1) in the text | “I have a nice balcony in my house” (0)  “I entered the balcony acting like a real Florence Nightingale” (1)  “I don’t like people with beards” (0)  “I don’t like growers of crops situated between nose and neck” (1) |
|  | Low-frequent words | Proportion of words in the text that belongs to the 20,000 most frequent Dutch words based on the SoNar corpus | Automatic coding (T-Scan);  Proportion scores (between 0 and 1);  Number of 20,000 most frequent words used divided by total number of words | “I am a man from Amsterdam and searching for a nice woman” (all underlined words are part of the 20,000 most frequent Dutch words, 1.00 indicates the proportion score)  “Apples, Archie Bunker, Börek, Berlin, Blues” (0.33) |
|  | Adjectives | Proportion of adjectives in the profile text | Automatic coding (T-Scan);  Proportion scores (between 0 and 1);  Number of adjectives in the text divided by total number of words in the text | “This sporty and intelligent man searches a smart partner” (0.27 indicates proportion score)  “50 years – cheeky – loving – masculine – wine – tapas” (0.43) |
|  | Adverbs | Proportion of adverbs in the profile text | Automatic coding (T-Scan);  Proportion scores (between 0 and 1);  Number of adverbs in the text divided by total number of words in the text | “rather positive than negative… rather Apple than Windows...” (0.25 indicates proportion score)  “This highly disciplined and always cheerful woman recently signed up for this site” (0.23) |
| Self-disclosure features | Number of words | Total number of words in a text | Automatic coding (LIWC);  Total number of words in a text |  |
|  | Number of self-disclosure statements | The amount of personal information a profile owner reveals about him/herself. This could also go in an indirect way (presenting personal information via an external source). This involves only information about the profile owner him- or himself and not the type of relationship (partner) the profile owner is looking for (see presence of looking-for clauses). | Manual coding;  Total number of self-disclosure statements in the text | “I’m a 50-year old man (all underlined words indicate one self-disclosure statement, i.e., 2 in this clause)  “I’m a funny and sweet 50-year old man” (4)  “Hobbies: volleyball, swimming, listening to music, dancing” (4) |
|  | Self-disclosure intimacy  (See [OSF file Details on Coding Procedure](https://osf.io/yns83/?view_only=5669e7863dce4b2ea2d3d0816f5e7689) for more information on this feature) | The extent to which the self-disclosure clause provides intimate information that is:  (1) low intimate: biographical, demographic and descriptive information without evaluative aspect (name, age, height)  (2) medium intimate: personal information with an evaluative aspect (personality, hobbies), or  (3) high intimate: personal information that has a normative or moral value (norms and values, secrets, wishes) | Manual coding;  Sum (1, 2, 3) score of the intimacy of the different clauses in the text | “I’m a man from Noord-Brabant” (low, scores 1)  “My dog is called Door” (low)  “I am a kind and fun person” (medium, scores 2)  “In my weekends, I often go to the gym” (medium)  “In my life, sharing is the keyword” (high, scores 3)  “I am very proud of what I have achieved in my life” (high)  “My life motto is: it’s all in a mindset” (high) |
|  | Presence of concrete self-disclosure | The occurrence of personal information that is specific and concrete as it evokes a clear imagery, for example by describing an assignable location, product, activity, period or moment. This could also involve information that specifies other information that is provided. | Manual coding;  Binary: absent (0) or present (1) in the text | “Coffee and a cracker with cheese or jam are essential in my morning ritual” (evokes a clear image of the writer’s breakfast)  “I don’t use a teabag twice”  “I love going for a walk at the coast” (specification of walking)  “I love cooking. Home-made sushi is my specialty” (specification of cooking) |
|  | I-references | Percentage of I-references in the profile text, that is, references to the self (e.g., ‘I’ and ‘me’ in English). In Dutch: ‘’k’, ‘ik’, ‘ikzelf’, ‘m’n’, ‘me’, ‘mezelve’, ‘mij’, ‘mijn’, ‘mijne’, ‘mijns’, ‘mijzelf’. | Automatic coding (LIWC);  Proportion scores converted to percentage scores (between 0 and 100);  Number of I-references in the text divided by total number of words in the text | “I often go to the gym” (16.67 indicates percentage score)  “Will you become mine?” (25.00)  “Online dating seemed a good idea to me, as I am a bit shy myself” (20.00) |
|  | Article use | Percentage of articles (i.e., ‘a’, ‘an’ or ‘the’ in English) used in the profile text. In Dutch: ‘de’, ‘het’, and ‘een’. | Automatic coding (LIWC);  Proportion scores converted to percentage scores (between 0 and 100);  Number of articles in the text divided by total number of words in the text | “Love to go for a walk” (16.67 indicates percentage score)  “Love to go for a walk at the coast” (22.22)  “Love to go for a walk at the coast on a sunny day” (23.08) |
| Perspective-taking features | Profile perspective | Whether the profile text is fully written from the writer’s own point of view (the own eyes) or also (partially) from another perspective:  (0) profile text that is fully written from the eyes of the profile owner (‘I’), or:  (1) profile text that is partially or fully written from another perspective than the profile writer’s perspective. This could also be a 3^rd^ person perspective (the own 3^rd^ person ‘(s)he’ or this (wo)man or from a poet, a narrator). | Manual coding;  Binary: text with full self-perspective (0) or no full self-perspective (1) | "Hi, I am new to this site, I will adjust my text later” (0)  “I am a nice who would like to meet a friendly woman” (0)  “This nice man would like to meet a sweet woman.” (1; 3^rd^ person)  "Is there more chance of a date with a suitable text? Or has the date more chance of a suitable man? My agenda is ready" (1)  “Ingredients: 3 spontaneous spoons of olive oil • 1 big humorous onion, frittered • 300g creative winter carrot, grated…” (1) |
|  | Presence of looking-for clauses | The occurrence of clauses that refer to information provided by the profile owner about what kind of relationship (partner) is sought | Manual coding;  Binary: absent (0) or present (1) in the text | “My partner should be intelligent and humorous” (1)  “I prefer to start with a date, and see what happens next” (1) |
|  | Question marks | Percentage of question marks in the text. | Automatic coding (T-Scan);  Proportion scores (between 0 and 1):  Number of question marks in the text divided by total number of words in the text | “Hi all! This is me. Who are you.” (0.00 indicates percentage score)  “Hi all! This is me. Who are you?” (0.33)  “Hi all, how are you? Are you ready to find love?” (1.00) |
|  | You-references | Percentage of you-references in the profile text, that is, references to the other person (e.g., ‘you’, ‘your’ in English). In Dutch: ‘ge’, ‘gij’, ‘gijzelf’, hullie’, ‘hulliejen’, ‘je’, ‘jezelf’, ‘jij’, ‘jijzelf’, ‘jou’, ‘jouw’, ‘jouwe’, ‘jouwer’, ‘jouzelf’, ‘jullie’, ‘oe’, ‘u’, ‘uw’, ‘uws’, ‘uzelf’ | Automatic coding (LIWC);  Proportion scores converted to percentage scores (between 0 and 100);  Number of you-references in the text divided by total number of words in the text | “I am looking for you” (20.0 indicates percentage score)  “Your sense of humor should be good” (14.29)  “You and I should just meet and maybe I’ll become yours” (16.67) |
